# Experimental confirmation of self-imaging effect between guided light and surface plasmon polaritons in hybrid plasmonic waveguides

**DOI:** 10.1038/s41598-022-22796-8

**Published:** 2022-10-26

**Authors:** Hiroyuki Okamoto, Shun Kamada, Kenzo Yamaguchi, Masanobu Haraguchi, Toshihiro Okamoto

**Affiliations:** 1 Department of Creative Technology Engineering, National Institute of Technology, Anan College, Anan, Tokushima 774-0017 Japan; 2 Advanced ICT Research Institute, National Institute of Information and Communications Technology, Kobe, Hyogo 651-2492 Japan; 3 Institute of Post-LED Photonics, Tokushima University, Tokushima, Tokushima 770-8501 Japan

**Keywords:** Nanophotonics and plasmonics, Optoelectronic devices and components

## Abstract

We fabricated a hybrid plasmonic device using self-imaging effect between guided light and surface plasmon polaritons in the hybrid plasmonic waveguide. The hybrid plasmonic device was fabricated by evaporating gold on the part of the silicon waveguide. Self-imaging was generated at the gold-covered section in the waveguide. Self-imaging of guided light and surface plasmon polaritons in hybrid plasmonic waveguides affect the output intensity of the hybrid plasmonic waveguide. The length of the hybrid plasmonic waveguide changes self-imaging conditions. We confirmed that the output intensity was affected by the length of the hybrid plasmonic waveguide. These findings contribute to the development of hybrid plasmonic devices and potentially improve integration density of hybrid photonic integrated circuits.

## Introduction

Recent technological advances in telecommunications have led to a rapid increase in communication traffic. To cope with this increase in traffic, optics are being used to increase the capacity of networks. However, it is difficult to fabricate integrated circuits of a practical size using only optical devices because they are much larger than electronic devices, owing to the diffraction limit of light^1–4^. Therefore, the use of surface plasmon polaritons (SPPs), which can be converted from optical energy and have no diffraction limit, is being considered for communication. However, SPPs have very high loss, making it difficult to construct devices or integrated circuits using only SPPs^5,6^. To address these issues, devices applying hybrid plasmonic structures that combine light and SPPs to effectively utilize the features of each to reduce losses have been reported^7–17^.

However, further simplification of the hybrid device structure is required to realize integrated circuits with high integration density^18–23^. The hybrid device structures previously reported have complex structures that consist of several portions. To incorporate these hybrid devices to photonic integrated circuits is not easy because the fabrication process is needed to several steps for fabricating these hybrid devices. In order to simplify the device structure, the devices that using self-imaging have been reported^24–28^. However, the devices using self-imaging induced by multi-mode interference requires to process a portion of the waveguide structure to control propagation modes. For example, the device structure using self-imaging we reported previously needed to process the depth of the device structure to control propagation modes^29^. We designed the device utilizing self-imaging effect between SPPs and the guided light in the hybrid plasmonic waveguide^30^. The device structure using self-imaging between SPPs and guided light was designed to propagate the fundamental mode of SPPs and that of guided light in the hybrid plasmonic waveguide. Then, a process to the waveguide only needs to evaporate gold on the waveguide to control propagation modes of the designed structure. The designed structure has thus an advantage of incorporating easily into photonic integrated circuits because the fabrication process of the designed structure is less than that of structures previously reported.

In this study, we designed and fabricated a hybrid plasmonic device and experimentally confirmed self-imaging of guided light and SPPs. Our device controls output by controlling self-imaging according to the length of the hybrid plasmonic waveguide. Accordingly, we fabricated several hybrid plasmonic waveguides of different lengths and evaluated the relationship between the length of the hybrid plasmonic waveguide and the output of light at 1300 nm to confirm self-imaging effect between the light waveguide and the SPPs in the hybrid plasmonic waveguide.

## Results and discussion

### Designed structure

**Figure 1:**
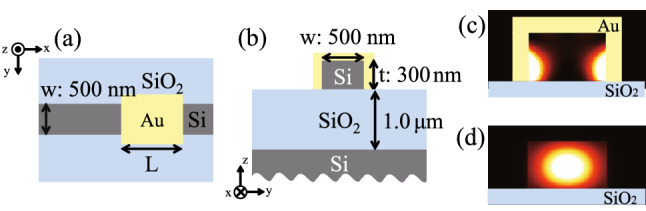
Schematics of the hybrid plasmonic waveguide structure: (**a**) the x-y plane view and (**b**) the y-z plane view. Electric field intensity distributions in the y-z plane at wavelength of 1300 nm: (**c**) SPPs in the hybrid plasmonic waveguide and (**d**) guided light in the optical waveguide.

Figure 1a, b show the designed structure. A specific length of a portion of the silicon waveguide is covered with gold. The gold-covered section is a hybrid plasmonic waveguide that combines an optical waveguide and a plasmonic waveguide, and a portion of the guided light propagating through the optical waveguide couples to the SPPs, such that the light and SPP propagate. Self-imaging occurs when light propagating in an optical waveguide interferes with SPP propagating in a plasmonic waveguide. With this structure, the length of the hybrid plasmonic waveguide is modified by changing the length of the gold covering, which affects the self-imaging in the waveguide. The output intensity is controlled by the self-imaging condition at the coupling between the hybrid plasmonic waveguide and the optical waveguide. The thickness of gold deposited on the silicon waveguide was 100 nm. The height (t) and width (w) of the optical waveguide and plasmonic waveguide were 300 nm and 500 nm, respectively, at which the fundamental modes of the guided light and SPPs can propagate. We calculated the effective refractive index of the fundamental modes for both SPPs and guided light at the wavelength of 1300 nm^31^. Figure 1c,d show electric field intensity distributions in the y-z plane at wavelength of 1300 nm for SPPs in the hybrid plasmonic waveguide and guided light in the optical waveguide, respectively. The effective refractive index is 3.20 for SPPs and 2.89 for guided light. The beat length of the self-imaging between guided light and SPPs is calculated using^24^

1 $$\begin{aligned} L_{B} = \pi /(\beta _{SPPs} - \beta _{Guided \, light}) \end{aligned}$$

where $$ {L_B}$$, $$\beta _{SPPs}$$, and $$\beta _{Guided \, light}$$ denote the beat length of the self-imaging between the SPPs and the guided light and the propagation constants of the SPPs and the guided light, respectively. The beat length of the designed structure was calculated using Eq. (1) to be 2100 nm.

### Characteristics of the hybrid plasmonic waveguide

**Figure 2:**
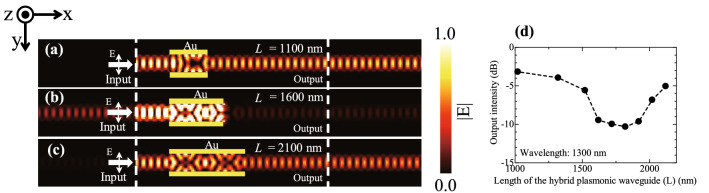
Profiles of electric field intensity at the centre of the hybrid plasmonic waveguide in the designed structure with (**a**) L = 1100 nm, (**b**) L = 1600 nm and (**c**) L = 2100 nm. The incident light wavelength is 1300 nm. The incident light has an electric field component in the y direction. (**d**) Output intensities as functions of L for the designed structure. The wavelength of incident light is 1300 nm.

Figure 2a,b and c show the electric field intensity distributions obtained using the finite difference time domain (FDTD) method for structures with gold lengths (L) of (a) 1100 nm, (b) 1600 nm, and (c) 2100 nm covering the silicon waveguide. The MIT electromagnetic equation propagation (MEEP) was used as a tool for the FDTD method^32^. The wavelength of the incident light was set to 1300 nm. For structures with L of 1100 nm (approximately half the beat length), and 2100 nm (the same length as the beat length), the electric field intensity distribution caused by self-imaging is concentrated in the centre of the waveguide at the switch from the hybrid plasmonic waveguide to the optical waveguide, and is coupled to the waveguide without reflection. However, for the structure of length 1600 nm, where L is approximately 0.75 times the beat length, self-imaging at the coupling between the hybrid plasmonic waveguide and the optical waveguide is concentrated at the sidewall of the waveguide and is reflected at the coupling, resulting in almost no coupling to the waveguide. This results in a decrease in the light intensity reaching the output port, showing that changing L affects self-imaging, which in turn affects the output light intensity. Figure 2d shows output intensities as functions of L for the designed structure. The output intensity decreases to about -10 dB when L ranges from 1600 to 1900 nm. The output intensity is however about from -4 to -5 dB when L is the beat length or 0.5 times the beat length.

### Fabricated structure

**Figure 3:**
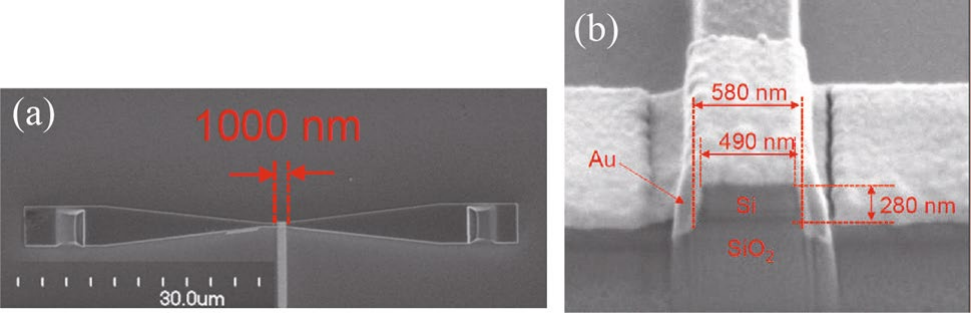
Scanning electron microscopy image of the fabricated structure with 1000 nm of the length of the hybrid plasmonic waveguide (L). (**a**) Image from the top and (**b**) a cross section of the hybrid plasmonic waveguide.

Figure 3a shows an image from the top and Fig. 3b a cross section of the hybrid plasmonic waveguide. A structure was fabricated with waveguide height t of 300 nm, waveguide width w of 500 nm, without deposited gold (structure of an optical waveguide only), and several structures with L varied in 100 nm steps between 1000 and 2000 nm. The input and output ports of the structure were machined into the tapered port shown in Fig. 3a to allow observation of the incoming and output light from the top.

### Characteristics of the trapezoidal waveguide structure

**Figure 4:**
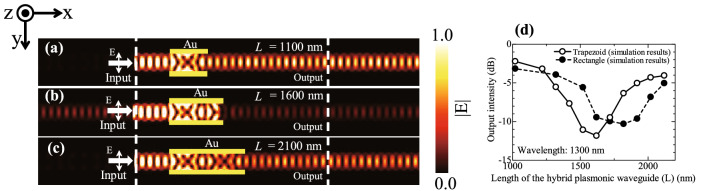
Profiles of electric field intensity at the centre of the hybrid plasmonic waveguide in the fabricated trapezoidal structure with (**a**) L = 1100 nm, (**b**) L = 1600 nm and (**c**) L = 2100 nm. The incident light wavelength is 1300 nm. The incident light has an electric field component in the y direction. (**d**) Output intensities as functions of L. Open circles show simulation results for the fabricated trapezoid structure. Closed circles show simulation results for the designed rectangular structure. The wavelength of incident light is 1300 nm.

Although the cross section of the waveguide was rectangular in the structure design, the cross section of the actual fabricated waveguide had a trapezoid form, as shown in Fig. 3b. To confirm the effect on output in the case of a trapezoid cross section, we evaluated a trapezoid waveguide structure of the same size as the fabricated structure using the FDTD method. The wavelength of the incident light was 1300 nm, the top and bottom of the trapezoid form were 490 nm and 580 nm, respectively, and the height was 280 nm. Figure 4a,b and c show the electric field intensity distributions obtained using the FDTD method for fabricated trapezoidal shape structures with gold lengths (L) of (a) 1100 nm, (b) 1600 nm, and (c) 2100 nm covering the silicon waveguide. Profiles of the electric field intensity of the fabricated trapezoidal shape structure are almost the same as profiles of the designed rectangle structure. The output intensity is decreased when L is 1600 nm. Output intensities are increased when L is 1100 and 2100 nm. Figure 4d shows the relationship between length L and output intensity for the structures with trapezoid and rectangular cross sections was determined by the FDTD method. The open circles (solid line) show the output intensity of the fabricated trapezoid structure and the solid circles (dashed line) show the output intensity of the designed rectangular structure. For the structure of L = 1000 nm, the output intensity was about -2 dB for both trapezoid and rectangular forms, and the output intensity was not significantly affected by changes in structure. However, when L was 1600 nm in the trapezoid structure, the output intensity became tiny at -12 dB. The output intensity increased as L exceeds 1600 nm. On the other hand, the rectangular structure showed almost no change in output intensity near -10 dB in the range of L from 1600 to 1900 nm. Thus, the trapezoid form of the structure shortens length L at which the output intensity reaches a minimum, and also narrows the range of length L at which the output intensity decreases and there is almost no change.

### Experimental confirmation of self-imaging effect

**Figure 5:**
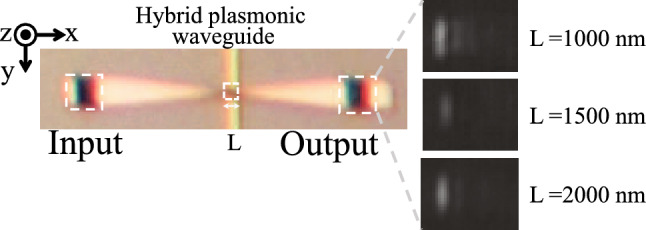
Optical microscope images of the fabricated structure and near-infrared camera images at the output port for different lengths of L = 1000, 1500, and 2000 nm.

Figure 5 shows optical microscope images of the hybrid plasmonic waveguide and near-infrared camera images at the output port for different lengths of L. The light output intensity at the output port is strong for the structure where L is 1000 nm (approximately half the beat length), and the structure where L is 2000 nm (approximately the same as the beat length). However, when L is 1500 nm (about 0.75 times the beat length), the output intensity at the output port is much lower than when L is 1000 or 2000 nm.

**Figure 6:**
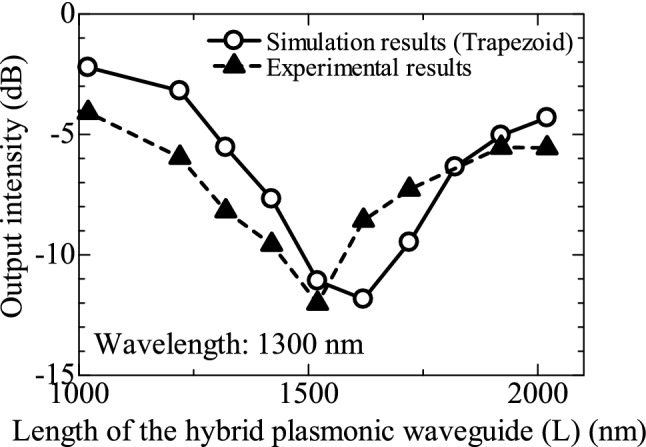
Output intensities as functions of L. The simulation results of the trapezoid structure same as the fabricated structure are shown by open circles (solid line), and the experimental evaluation results are shown by solid triangles (dashed lines).

Figure 6 shows how light intensity at the output port changes as a function of length L in the range of 1000 to 2000 nm. The intensity at the output port was analysed by ImageJ^33^ from the near-infrared camera image, 0.0 dB was made the same as the output intensity at the output port of the optical waveguide structure without Au deposition, and the output intensity at each L was determined from the image. FDTD simulation results for the same trapezoid structure as the fabricated structure are shown by open circles (solid line), and the experimental evaluation results are shown by solid triangles (dashed lines). The experimental results show that the output light intensity reaches a minimum of -12 dB at an L of around 1500 nm, while the simulation results show that the output light intensity reaches a minimum of -12 dB at an L of around 1600 nm. In this way, we confirmed the effect of L, the length of the hybrid plasmonic waveguide we devised, on output intensity. This result confirms the self-imaging effect between guided light and SPPs in hybrid plasmonic waveguides. Although there is a discrepancy of about 100 nm between the experimental and simulation results in the length of L at which the output intensity reaches a minimum, the relationship between L and the change in output intensity is almost in agreement in the experimental and simulation results. The simulated structure is trapezoidal in form to match the fabricated structure, which corrects for the 100 nm dip when compared to the simulation results of the originally designed rectangular structure. However, the simulated structure is not exactly the same as the fabricated structure, and the deviation between these structures may be the cause of this error.

## Conclusion

We fabricated hybrid plasmonic devices of different lengths and evaluated their output intensity with incident light of wavelength 1300 nm to confirm the effect of self-imaging of guided light and SPPs in hybrid plasmonic waveguides. At 1000 nm (a hybrid plasmonic waveguide length about half the beat length), and at 2000 nm (about the same as the beat length), the electric field intensity distribution owing to self-imaging at the coupling between the hybrid plasmonic waveguide and the optical waveguide is strong in the centre area, smoothly coupling to the optical waveguide and with no reduction in output intensity. On the other hand, when the length of the hybrid plasmonic waveguide is 1500 nm (about 0.75 times the beat length), the electric field intensity distribution by self-imaging in the coupled area is strong near the sidewall of the hybrid plasmonic waveguide, there is reflection at the coupling with the optical waveguide, and a decrease in output intensity was confirmed.

The application of self-imaging using guided light and surface plasmon polaritons confirmed in this study. The findings of this study will enable the development of hybrid plasmonic devices, which could significantly contribute to improving the integration density of hybrid photonic integrated circuits and increasing communication capacity.

## Methods

### Fabrication process

**Figure 7:**
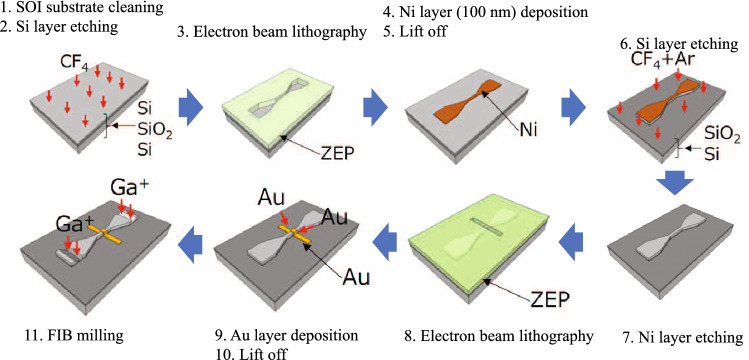
Fabrication processes of the hybrid plasmonic waveguide.

Figure 7 shows the fabrication process of the hybrid plasmonic waveguide. First, etching is performed to make the thickness of the Si layer on the device surface of the SOI substrate 300 nm. Next, the Si waveguide structure and taper are fabricated using electron beam lithography. After fabrication of the Si waveguide structure, Au is deposited on the fabricated Si waveguide structure using electron beam lithography in order to fabricate the hybrid plasmonic waveguide. Au is deposited from an oblique direction so that it can be deposited on the side surface of the Si waveguide. Finally, a tapered portion is machined by FIB to fabricate input/output ports.

### Experimental setup

**Figure 8:**
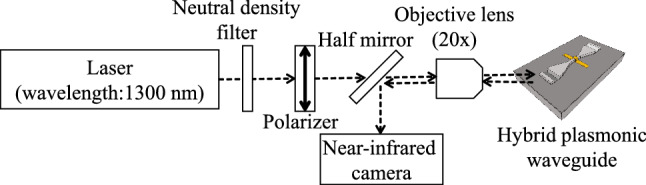
Schematic of the experimental setup to evaluate the fabricated structure. The wavelength of incident light is 1300 nm.

The output intensity of the fabricated structures was evaluated using the setup shown in Fig. 8. A laser with an emission wavelength of 1300 nm was used as the input light source, and an objective lens was used to illuminate the input port with a spot size of 5 $\mu$m. The intensities at the output port of the hybrid plasmonic waveguide were recorded by a near-infrared camera through an objective lens and a half mirror.

## Data Availability

The datasets used and/or analysed during this study available from the corresponding author on reasonable request.
